# Availability, quality and price of produce in low-income neighbourhood food stores in California raise equity issues

**DOI:** 10.1017/S1368980018000058

**Published:** 2018-03-15

**Authors:** Wendi Gosliner, Daniel M Brown, Betty C Sun, Gail Woodward-Lopez, Patricia B Crawford

**Affiliations:** 1 Nutrition Policy Institute, University of California Division of Agriculture and Natural Resources, 2115 Milvia Street, Berkeley, CA 94704, USA; 2 Contra Costa Health Services, Martinez, CA, USA; 3 Golden Gate National Parks Conservancy – Institute at the Golden Gate, San Francisco, CA, USA

**Keywords:** Fruits and vegetables, Produce availability, quality, price, Health disparities, equity, Retail food stores, SNAP-Ed, low-income

## Abstract

**Objective:**

To assess produce availability, quality and price in a large sample of food stores in low-income neighbourhoods in California.

**Design:**

Cross-sectional statewide survey.

**Setting:**

Between 2011 and 2015, local health departments assessed store type, WIC (Supplemental Nutrition Program for Women, Infants, and Children)/SNAP (Supplemental Nutrition Assistance Program) participation, produce availability, quality and price of selected items in stores in low-income neighbourhoods. Secondary data provided reference chain supermarket produce prices matched by county and month. *t* Tests and ANOVA examined differences by store type; regression models examined factors associated with price.

**Subjects:**

Large grocery stores (*n* 231), small markets (*n* 621) and convenience stores (*n* 622) in 225 neighbourhoods.

**Results:**

Produce in most large groceries was rated high quality (97 % of fruits, 98 % of vegetables), but not in convenience stores (25 % fruits, 14 % vegetables). Small markets and convenience stores participating in WIC and/or SNAP had better produce availability, variety and quality than non-participating stores. Produce prices across store types were, on average, higher than reference prices from matched chain supermarkets (27 % higher in large groceries, 37 % higher in small markets, 102 % higher in convenience stores). Price was significantly inversely associated with produce variety, adjusting for quality, store type, and SNAP and WIC participation.

**Conclusions:**

The study finds that fresh produce is more expensive in low-income neighbourhoods and that convenience stores offer more expensive, poorer-quality produce than other stores. Variety is associated with price and most limited in convenience stores, suggesting more work is needed to determine how convenience stores can provide low-income consumers with access to affordable, high-quality produce. WIC and SNAP can contribute to the solution.

The importance of fruit and vegetable consumption to human health has been well established. Fruit and vegetable intake is associated with reductions in chronic diseases including heart disease, stroke and some cancers^(^ ^1^ ^,^ ^2^ ^)^. However, in the USA, less than 10 % of the population consumes adequate fruits and vegetables consistent with dietary recommendations^(^ ^3^ ^,^ ^4^ ^)^. Socio-economic disparities in fruit and vegetable consumption, while not new, have increased; for example, a 2014 study found the gap in Alternate Healthy Eating Index (AHEI) scores between adults of low and high socio-economic status had grown from 3·9 points in 1999–2000 to 7·8 points in 2009–2010 (maximum AHEI score=110; mean 2009–2010 adult score=46·8)^(^ ^5^ ^)^.

A number of possible mechanisms have been suggested as contributing factors to these disparities. One area that has received widespread attention in recent decades is the association of access to fresh fruits and vegetables in the neighbourhood food retail environment with health behaviours and outcomes^(^ ^6^ ^–^ ^8^ ^)^. Studies have found that children living near food stores with lower prices of fruits and vegetables have lower increases in BMI^(^ ^9^ ^)^, and that quality, selection and convenience in the food retail environment in low-income neighbourhoods are predictors of fruit and vegetable consumption^(^ ^10^ ^)^. Further, studies of low-income consumers suggest that both food quality and price are key factors in shopping decisions^(^ ^11^ ^,^ ^12^ ^)^.

While many studies have examined whether residents of low-income communities have poorer access to healthy foods, fewer have examined the quality and/or price of healthy fruits and vegetables in these neighbourhoods. Most recent studies have relied upon relatively small samples of stores and the findings have been somewhat inconsistent. A 2012 systematic review of the evidence reported that six of ten studies conducted in the USA found less availability of healthy foods in low-income or high-minority communities; only two studies reported lower-quality food items in low-income neighbourhoods^(^ ^13^ ^)^. Food price results in that systematic review also were inconsistent: six studies found that healthy foods were cheaper at larger compared with smaller grocery stores; three found healthy foods to be more expensive at convenience stores compared with grocery stores and supermarkets; and two studies found higher prices of healthy foods in low-income neighbourhoods across store types, while two others found no significant price differences when comparing supermarkets across low- and high-income communities^(^ ^14^ ^)^. One California study conducted over a decade ago evaluated the cost of a fruit and vegetable market basket in twenty-five supermarkets and found that prices were lower in low-income neighbourhoods than in middle- and high-income neighbourhoods^(^ ^15^ ^)^. While the latter study included bulk, independent and chain supermarkets, the assessment of prices by neighbourhood was not adjusted for store type. Currently, a good deal of community intervention work focuses on increasing access to fruits and vegetables in smaller neighbourhood stores^(^ ^16^ ^,^ ^17^ ^)^; however, few small food store interventions address price^(^ ^18^ ^,^ ^19^ ^)^.

Of particular interest are opportunities for the Supplemental Nutrition Assistance Program (SNAP) and the Supplemental Nutrition Program for Women, Infants, and Children (WIC) to influence the food retail environments of income-constrained shoppers. Studies have shown that many small and mid-sized food stores participating in SNAP carry a limited selection of healthy foods^(^ ^20^ ^)^. One recent study in New Jersey found that participation in WIC, but not SNAP, was associated with better availability of healthy foods in corner stores^(^ ^21^ ^)^. Studies describing differences in food availability in WIC- and/or SNAP-accepting stores are relatively limited.

In response to concerns about disparities in fruit and vegetable access and consumption, the Supplemental Nutrition Program Education (SNAP-Ed) efforts of the California Department of Public Health prioritized improving fruit and vegetable access in the food retail setting. In establishing this intervention focus area, the California Department of Public Health developed and reliability tested a community-driven neighbourhood food environmental assessment protocol called Communities of Excellence in Nutrition, Physical Activity, and Obesity Prevention (CX^3^) to help local health departments assess low-income neighbourhood food environments and identify retailers with whom to work to improve healthy food access^(^ ^22^ ^)^. The present paper describes the large sample of grocery stores, small markets and convenience stores in low-income neighbourhoods that participated in CX^3^ baseline assessments from 2011 to 2015. The purpose of the current cross-sectional study was to determine the extent to which fruits and vegetables are available in stores in low-income neighbourhoods across the state and to assess the degree to which availability, quality and price vary by store type and federal food programme participation. Recognizing the price sensitivity of income-constrained consumers, we also examined the store characteristics most associated with lower produce prices. Further, we sought to assess whether produce items in our sampled stores were priced higher or lower than average chain supermarket store prices in the same counties during the same month.

## Methods

### Sample

The data were collected as part of a retail food intervention project of the California Department of Public Health, Nutrition Education and Obesity Prevention Branch with SNAP-Ed funding from the US Department of Agriculture. The project aimed to prepare local community leaders to identify issues in the food retail environment that they could work to improve and focused on partnership development and stakeholder engagement^(^ ^22^ ^)^.

Local health department leaders selected low-income neighbourhoods for inclusion in the data collection efforts if the neighbourhoods were being considered for potential interventions. The local community was responsible for defining the neighbourhood boundaries based upon local conditions and residents’ perceptions. Eligible stores were required to be located in a SNAP-Ed eligible census tract (defined as a tract in which at least 50 % of the population is at or below 185 % of the federal poverty level^(^ ^23^ ^)^). In some cases, the identified neighbourhoods included a limited number of census tracts in a large city, while in other cases they included an entire town. Because census tracts and neighbourhood boundaries did not always align, some neighbourhoods selected by local leaders included stores in eligible and ineligible census tracts. Stores in ineligible census tracts were excluded. Local leaders were instructed to collect data in all eligible food retail stores in which the primary product sold was food. Liquor stores, bakeries, cafés, restaurants and other similar establishments were excluded.

### Data collection

Data collection occurred on a rolling basis over four years, until all health departments in California participating in the Nutrition Education and Obesity Prevention Branch SNAP-Ed programme had an opportunity to participate. The CX^3^ Food Availability and Marketing Survey was used to assess community food store environments. In-person trainings were offered annually and included store visits in which data collectors had an opportunity to complete the survey form and compare their responses against those of the trainer. In some cases, local staff who attended the State training trained additional data collectors, including community partners, community residents and students. Prior to completing the on-site assessments, data collectors were trained to gain permission from the store manager. Store assessments were completed by single or pairs of data collectors and took approximately 30 min per store to complete. With a few exceptions, stores in a single neighbourhood were surveyed within the same week. Data were double entered by professional data-entry staff.

### Store assessment measures

#### Store characteristics

Local leaders obtained initial descriptions of store characteristics from Geographic Information Systems store lists using annually updated data from state databases as well as data purchased from Dunn & Bradstreet. All store information was verified during on-site visits. Store type was identified as supermarket chain stores, large grocery stores (≥4 cash registers but not large chains), small markets (<4 registers but selling a range of foods, including fresh meat), convenience stores (selling food items and snacks but not a complete range of foods and no fresh meat; may sell gas) and other (included WIC-only stores, dollar stores and drug stores/pharmacies); this store type assessment was verified during on-site visits. Store participation in the US Department of Agriculture’s WIC and/or SNAP programme(s) was obtained from the Geographic Information Systems and verified during on-site visits or by telephone as needed.

#### Fruit and vegetable availability and quality

The survey instrument included an item that assessed whether produce was sold or not. In stores where produce was available, data collectors recorded the variety of fresh fruits available (none; 1–3 types; 4–6 types; ≥7 types) and repeated the assessment for fresh vegetables. Similarly, the quality of fruits was assessed on a scale of 1–4 (1=most of the options are brown, bruised, overripe and/or wilted; 4=all or most of the options appear fresh, without soft spots, excellent colour) and was repeated for fresh vegetables. These were coded as binary variables, with ‘high-quality’ fruits or vegetables indicating the top two categories.

#### Cost of fruits and vegetables

A single price for a small selection of fruits and vegetables commonly found in the USA (apples, bananas, oranges, carrots, tomatoes, broccoli and cabbage) was recorded. The recorded prices were the cheapest price per pound (0·454 kg) for each type of fruit or vegetable; thus, if a store carried many varieties of apples, the price recorded was the least expensive apple in the store. When prices per pound were not available, the price per package or bunch was recorded along with the weight of the bunch. In cases where only price per piece was available, that was recorded, and a US Department of Agriculture database of standard weights was used to convert to price per pound. Mean prices were not reported for broccoli and cabbage in convenience stores, as fewer than thirty stores in this category had recorded prices for these items.

To assess the store’s produce price against a price point benchmarked to geography and season, retail scanner data from chain supermarkets in the county were purchased from FreshLook Marketing Group. FreshLook data provided actual retail prices of perishable items sold at twenty-seven supermarket chains (including Safeway, Target, Nob Hill Foods, Food4Less, Raley’s, Vons, Walmart and others) in the sampled counties and were obtained to match the data collection month in each county. When same-month observations were not available, average in-county lowest prices across all months were used as the comparison group.

### Analyses

The 1474 stores identified as large groceries (includes supermarket chain stores and large grocery stores), small markets and convenience stores were included in the analyses. The fifty-three (4 %) excluded stores (including e.g. dollar stores, WIC-only stores and butcher’s shops) did not fit any of the defined store types.

Series of paired tests were performed between each of the store types to determine whether key variables differed by type. Three tests were performed for each variable, comparing grocery stores with small markets, grocery stores with convenience stores, and small markets with convenience stores. Comparisons of binary store characteristics between store types were done using paired two-sample tests of equality of proportions and continuous measures using ANOVA to estimate the studentized sample range differences. For all analyses, tests were deemed significant if they rejected the null hypothesis of coming from the same population, after controlling for test-specific family-wide error rate, with a *P* value of 0·05.

To calculate the reference average lowest price for produce items, lowest prices were drawn from the stores in the purchased data set matched to the month and county in which sampled store observations were completed. Relative price difference was calculated as the difference between the lowest sampled store price and the average in-county, in-month chain supermarket lowest price, expressed as a percentage of the average county chain supermarket lowest price. The average relative price difference across produce types was taken as the average of the relative prices of any of the five most common produce items present in the store (apples, bananas, oranges, carrots and tomatoes). We fit ordinary least-squares regression models, performing the regression of this average relative price *v*. a vector of store characteristics based upon variables in the data set that were selected *a priori*. These characteristics included: whether the store participated in the SNAP or WIC programme, whether the store was located within half a mile (0·8 km) of a school, whether the fruits and vegetables were rated high quality, and whether the store carried four or more types of fruits or four or more types of vegetables. A fixed effect was included to control for the county in which the stores were located. Regressions were run using the ‘lfe’ package and all calculations were performed using R version 3.2.2.

## Results

**Fig. 1:**
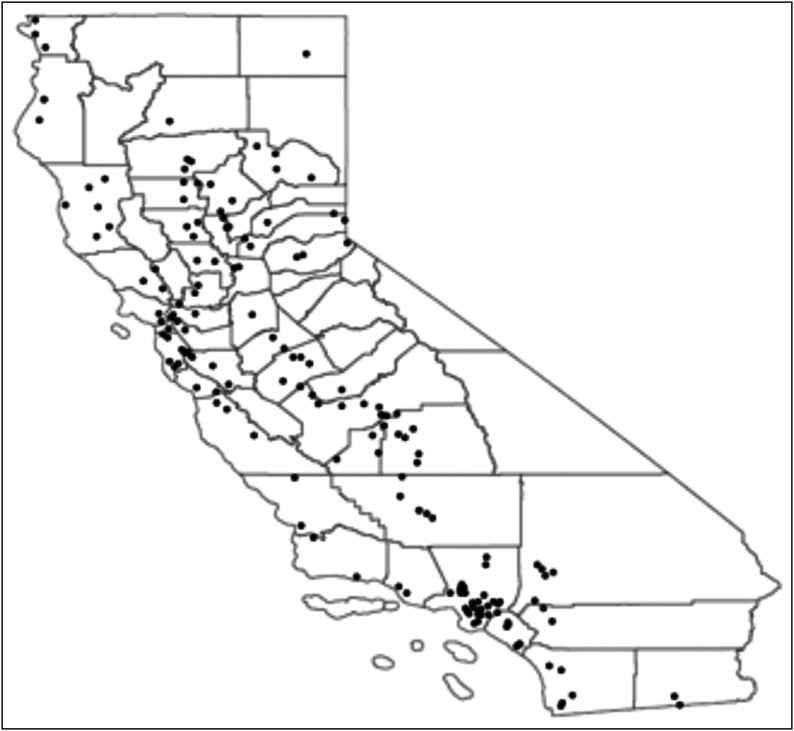
Locations in which stores included in the CX^3^ (Communities of Excellence in Nutrition, Physical Activity, and Obesity Prevention) sample are situated; cross-sectional statewide survey in low-income neighborhoods in California, 2011–2015

The final sample included 1474 stores in 470 unique census tracts within 225 low-income neighbourhoods in cities and towns across forty-four California counties (Fig. 1). All large grocery stores sold fresh produce and nearly all of those had a wide variety of high-quality offerings (Table 1). Most (80 %) small markets also sold fresh fruits and vegetables, but fewer had a wide variety and high quality compared with grocery stores. Fewer than half (41 %) of convenience stores sold produce; of those that did, only a small minority had a selection of at least four or more fruits (16 %) or vegetables (12 %), and few stores’ selections were rated high quality (25 % for fruits, 13 % for vegetables). Nearly all large grocery stores (95 %) and most small markets (79 %) and convenience stores (67 %) accepted SNAP benefits. Most large grocery stores (83 %) accepted WIC, but few small markets (34 %) or convenience stores (11 %) did. We observed significant differences between each of the store types for all variables examined, except the proximity to a school.

**Table 1:** Number and proportion of stores in the sample with selected characteristics; cross-sectional statewide survey in low-income neighbourhoods in California, 2011–2015

	Large groceries (*n* 231)		Small markets (*n* 621)		Convenience stores (*n* 622)		
	*n*	%	*n*	%	*n*	%	Significance*
Sells produce	230	100	497	80	257	41	A, B, C
≥4 Fruits	227	98	341	55	100	16	A, B, C
≥4 Vegetables	229	99	391	63	73	12	A, B, C
High-quality fruits	223	97	360	58	157	25	A, B, C
High-quality vegetables	226	98	368	59	84	14	A, B, C
Accepts SNAP	219	95	491	79	417	67	A, B, C
Accepts WIC	191	83	211	34	69	11	A, B, C
Half a mile (0·8 km) from a school	161	70	485	78	477	77	A

SNAP, Supplemental Nutrition Assistance Program; WIC, Supplemental Nutrition Program for Women, Infants, and Children.

* The significance column indicates the significance (at *P*<0·05) of two-way tests of the hypothesis that the proportions of interest differ between store type categories. The letter A indicates that ‘large groceries’ and ‘small markets’ have different proportions, B that ‘large groceries’ and ‘convenience stores’ differ, and C that ‘small markets’ and ‘convenience stores’ differ.

WIC and/or SNAP participation was associated with better quality and variety of produce (Table 2). Small markets and convenience stores participating in both WIC and SNAP were significantly more likely to sell any fresh produce and to have a wider variety and higher-quality produce items than stores not participating in either programme. SNAP-only small markets had significantly better availability, variety and quality of fresh produce than stores not participating in SNAP or WIC, and SNAP-only convenience stores were significantly more likely to sell fresh produce (43 % *v.* 29 %), carry four or more fruits (15 % *v.* 8 %) and carry fruits rated high quality (28 % *v.* 16 %) than convenience stores not participating in SNAP or WIC. Similar analyses could not be completed for large grocery stores, since nearly all of them participated in SNAP and WIC.

**Table 2:** Proportion of small markets and convenience stores in the sample meeting selected availability and quality criteria, as well as relative price differences*, by federal food programme participation status; cross-sectional statewide survey in low-income neighbourhoods in California, 2011–2015

	Small markets				Convenience stores			
	Neither WIC or SNAP (*n* 108)	SNAP only (*n* 302)	Both (*n* 211)		Neither WIC or SNAP (*n* 196)	SNAP only (*n* 357)	Both (*n* 69)	
	%	%	%	Significance†	%	%	%	Significance†
Sells fresh produce	68	76	97	A, B, C	29	43	71	A, B, C
≥4 Fruits	68	76	97	A, B, C	8	15	46	A, B, C
≥4 Vegetables	44	56	82	A, B, C	5	8	48	A, B, C
High-quality fruits	46	50	75	A, B, C	16	28	38	A, B, C
High-quality vegetables	43	54	75	A, B, C	8	13	32	A, B, C
Relative price differences								
Apples	71	55	48		122	105	111	
Bananas	43	44	33		132	127	62	B, C
Oranges	18	29	26		108	127	68	C
Carrots	39	39	47		110	108	97	
Tomatoes	−5	14	1	A, C	2	24	30	
Broccoli	0	8	7		–	–	–	–
Cabbage	−6	9	−8		–	–	–	–
Five-item average	40	39	33		121	106	74	B, C

WIC, Supplemental Nutrition Program for Women, Infants, and Children; SNAP, Supplemental Nutrition Assistance Program.

* Relative price differences are the difference between the observed lowest store price and the average lowest price for chain supermarkets in the same county that month, expressed as a percentage of the average county chain supermarket lowest price.

† The significance column indicates the significance (at *P*<0·05) of two-way tests of the hypothesis that the proportions differ. The letter A indicates that the ‘neither’ and ‘SNAP only’ groups differ, B that the ‘neither’ and ‘both’ groups differ, and C that the ‘both’ and ‘SNAP only’ groups differ.

In the sampled stores, the average recorded lowest prices for fruits and vegetables were lowest in large grocery stores for four of the items recorded (apples, bananas, carrots, broccoli) and lowest in small markets for three (oranges, tomatoes, cabbage; Table 3). Convenience store prices were higher than those in both large grocery stores and small markets for apples, oranges, bananas and carrots, but only higher than those in small markets for tomatoes (too few stores sold broccoli and cabbage to allow for meaningful inclusion).

**Table 3:** Average lowest price per pound (0·454 kg) and relative price difference* of seven produce items in the sample by store type; cross-sectional statewide survey in low-income neighbourhoods in California, 2011–2015

	Large groceries					Small markets					Convenience stores					
		Average lowest price ($US)			Relative price difference		Average lowest price ($US)			Relative price difference		Average lowest price ($US)			Relative price difference	Relative price test
Item	*n*	Mean	Range	sd	(%)	*n*	Mean	Range	sd	(%)	*n*	Mean	Range	sd	(%)	significance †
Apples	218	1·17	0·66–2·99	0·39	44	312	1·18	0·63–2·59	0·37	53	130	1·58	0·69–2·80	0·50	110	B, C
Bananas	218	0·70	0·49–1·49	0·16	15	336	0·85	0·49–2·19	0·31	39	150	1·30	0·49–2·19	0·46	115	A, B, C
Oranges	200	0·95	0·48–1·49	0·40	35	249	0·88	0·47–2·20	0·41	27	88	1·36	0·50–2·20	0·59	107	B, C
Carrots	179	0·84	0·50–2·14	0·33	33	242	0·91	0·50–2·20	0·37	43	24	1·22	0·50–2·00	0·58	102	B, C
Tomatoes	197	1·21	0·59–2·66	0·45	13	311	1·12	0·59–2·79	0·41	6	57	1·21	0·69–2·19	0·38	23	C
Broccoli	199	1·30	0·68–3·19	0·49	1	142	1·33	0·68–3·18	0·56	6	–	–	–	–	–	
Cabbage	172	0·74	0·38–1·59	0·27	0	261	0·66	0·34–1·50	0·26	−1	–	–	–	–	–	

* Relative price differences are the difference between the observed lowest store price and the average lowest price for chain supermarkets in the same county that month, expressed as a percentage of the average county chain supermarket lowest price.

† The test significance column indicates the significance (at *P*<0·05) of two-way tests of the hypothesis that the relative price differences differ between store type categories. The letter A indicates that ‘large groceries’ and ‘small markets’ stores have different relative price differences, B that ‘large groceries’ and ‘convenience stores’ differ, and C that ‘small markets’ and ‘convenience stores’ differ.

To compare prices across store type we used ‘relative price difference’, the difference between the average lowest prices in our sample and the county average lowest prices in chain supermarkets, expressed as a percentage of the average county lowest price in chain supermarkets. Overall, all store types in our sample had higher lowest prices for the observed produce items than the average lowest prices available in the reference group of chain supermarkets (Table 3). The relative price difference for the produce items studied ranged from 0 % to 44 % higher for large groceries, from −1 % to 53 % higher for small markets and from 23 % to 115 % higher for convenience stores. Mean relative convenience store produce prices were substantially higher than the mean relative prices in large groceries and small markets.

**Fig. 2:**
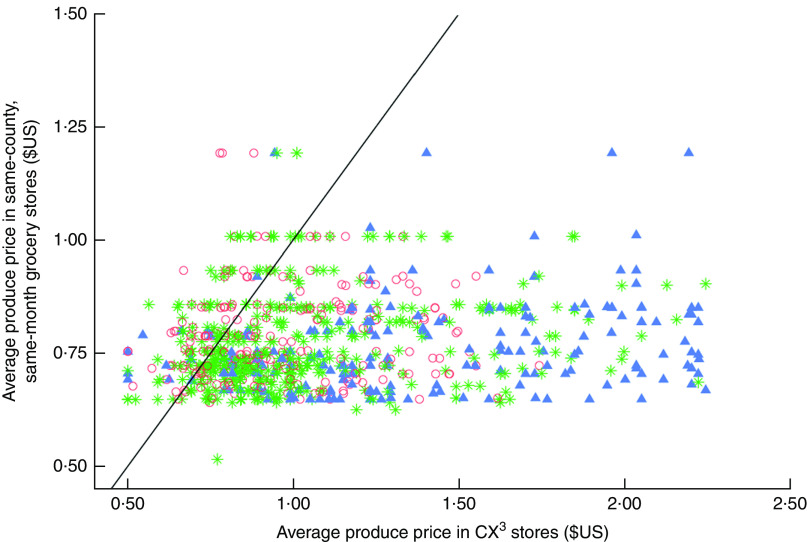
(colour online) Average lowest price of seven common fruits and vegetables in the sample stores, by store type (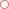, supermarkets/large groceries; 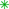, small markets; 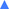, convenience stores), compared with average lowest price of the items in chain supermarket stores in the same county and the same month; cross-sectional statewide survey in low-income neighborhoods in California, 2011–2015. Diagonal line indicates where points would lie if average price in sampled stores and average price in same-county, same-month grocery stores were equal.

While the relative price differences for large grocery stores in our sample were smaller than the other store types, even the large grocery stores in our sample on average sold produce at higher prices than the reference county average in chain supermarkets (27 % higher relative price difference). A few stores of each type (large groceries, small markets, convenience stores) had average lowest prices for produce that were lower than the same-month county average (see stores to the left of the diagonal line in Fig. 2) but far more stores had higher average lowest prices.

Few significant relationships were observed between store produce prices and SNAP or WIC participation. In small markets, the only significant relationship observed was that relative tomato prices were significantly higher in SNAP-only small markets than in both non-participating and WIC+SNAP stores (14 % of county average *v*. −5 % and 1 %, respectively). In convenience stores, the combined average relative price of the five most commonly measured produce items was significantly lower in WIC+SNAP convenience stores compared with both other groups (74 % higher *v.* 106 % in SNAP-only stores and 121 % in stores not participating in SNAP or WIC). Additionally, in convenience stores, relative banana prices were significantly lower in WIC+SNAP stores than in other stores (62 % higher *v.* 127 % in SNAP-only stores and 132 % in stores not participating in SNAP or WIC); and oranges were significantly cheaper in WIC+SNAP stores than in in SNAP-only stores (68 % higher *v.* 127 % higher).

Regression models estimated that across all store types, the most significant predictor of price was whether a store carried four or more fruits or vegetables or not (Table 4). In the combined model including all store types, carrying more than four types of fruits and vegetables were both significantly associated with lower prices, a 17 % relative reduction (95 % CI −27, −7 %) for fruit and an identical 17 % relative reduction (95 % CI −29, −6 %) for vegetables. In small markets carrying four or more types of fruit, relative prices were 25 % cheaper compared with stores carrying fewer than four types of fruit (95 % CI −37, −14%). This same relationship was observed in convenience stores, where prices were 11 % cheaper in stores carrying four or more types of fruit, although the relationship was not statistically significant (95 % CI −43, 20 %). Average prices were 1·7 % cheaper in small markets (95 % CI −16, 13 %) and 38 % cheaper in convenience stores carrying more than four types of vegetables (95 % CI −69, −7 %).

**Table 4:** Parameter estimates and 95 % CI from regression analyses predicting the average relative price of five produce items (apple, bananas, oranges, carrots and tomatoes) as a function of store characteristics among three groups of sampled stores (all stores, small markets and convenience stores); cross-sectional statewide survey in low-income neighbourhoods in California, 2011–2015

	All stores		Small markets		Convenience stores	
	*β*	95 % CI	*β*	95 % CI	*β*	95 % CI
Accepts SNAP	−3·2	−12, 5·5	−1·8	−11·5, 7·9	4·8	−33·3, 42·9
Accepts WIC	−0·3	−7·0, 6·4	2·1	−6·0, 10·2	20·1	−7·7, 47·9
Half a mile (0·8 km) from a school	−8·5	−16·1, −0·9	−10·8	−20·7, −0·8	−6·0	−43·1, 31·1
High-quality fruits	2·5	−7·7, 12·7	−3·7	−14·9, 7·4	4·7	−39·9, 49·3
High-quality vegetables	−0·7	−11·2, 9·8	0·7	−11·1, 12·4	−1·2	−42·1, 39·7
≥4 Fruits	−17·3	−27·4, −7·1	−25·4	−37·2, −13·6	−11·4	−43·2, 20·4
≥4 Vegetables	−17·3	−28·7, −5·9	−1·7	−16·1, 12·7	−38·1	−69, −7·2
Small market	3·9	−3·5, 11·3	–	–	–	–
Convenience store	28·0	16·8, 39·2	–	–	–	–

SNAP, Supplemental Nutrition Assistance Program; WIC, Supplemental Nutrition Program for Women, Infants, and Children.

Prices for the five produce items were lower in the full sample of stores and in small markets located within half a mile of a school, but no relationship between distance from school and price was observed in convenience stores. No clear relationship was observed between fruit or vegetable quality and price. No significant quality-related price differences were observed in the full sample of stores or small markets. Overall, in the adjusted model, store type was significantly associated with produce price only for convenience stores, where the five-item average was 28 % higher (95 % CI 17, 39 %).

## Discussion

Within our large sample of stores in low-income neighbourhoods in California, we find that produce availability, variety and quality are best in large grocery stores, followed by small markets, and poorest in convenience stores. While most large grocery stores and small markets in our sample sold produce that was rated high quality, the selection of fruit was rated high quality in only 25 % of convenience stores and vegetables in only 14 % of convenience stores. At the same time, convenience stores that sold produce had substantially higher prices than large groceries or small markets.

Importantly, we find higher average store lowest prices in the low-income communities we assessed for the produce items examined (apples, bananas, oranges, carrots and tomatoes) compared with the county average chain supermarket prices in the same county in the same month across all store types, even in large grocery stores. While it might be expected that prices in chain supermarkets would be lower than in smaller stores, this large sample of stores in low-income neighbourhoods across California provides evidence to suggest that low-income residents who shop for food in their neighbourhoods may pay more, on average, for produce regardless of the type of store in which they shop. This is the largest sample of stores in recent decades to assess the relationship between neighbourhood sociodemographic characteristics and fruit and vegetable prices adjusted for store type. These findings are consistent with some earlier studies^(^ ^13^ ^)^ but contradict the findings of a 2007 study of California food stores that looked at a broader basket of foods^(^ ^15^ ^)^.

Additionally, we find that a wider variety of produce items is associated with lower prices within store type. Other studies have suggested that efforts to promote healthy eating should support retailers to expand their selection of healthy foods^(^ ^24^ ^)^. While the literature on retail interventions to improve diet has not been robust, some evidence does suggest that a wider variety of fruits and vegetables in corner stores is significantly associated with the odds of a customer purchasing those items, and the likelihood of purchasing was even stronger for customers participating in SNAP than non-participants^(^ ^25^ ^)^. Future studies should assess whether increasing store produce variety helps stores to offer more competitive prices and whether this leads to increased purchases, particularly among SNAP participants.

In our sample, SNAP and WIC programme participation among small markets and convenience stores was associated with better availability of produce overall as well as better variety and quality of fruits and vegetables. Consistent with earlier studies^(^ ^20^ ^)^, our analysis found that many convenience stores participating in SNAP fail to provide a wide variety of fruits and vegetables; but contrary to the previous study, our results find that SNAP stores perform better than stores not participating in SNAP or WIC. Consistent with a 2016 study of corner stores^(^ ^21^ ^)^, our data find that WIC participation (along with SNAP) of small groceries and convenience stores is more highly associated with produce availability and quality than SNAP participation alone or non-participation in either programme. Recently, efforts to revise SNAP retailer standards were undertaken at the federal level^(^ ^26^ ^)^. Our results suggest the programme (prior to the revisions) may already have played a role in encouraging stores, or attracting stores more willing, to offer fruits and vegetables. Given the poor variety of produce offered in SNAP-participating convenience stores in the present study, SNAP could provide a mechanism for increasing the variety and quality of produce in these stores, which could potentially reduce price.

Our study has a number of limitations. The cross-sectional design limits our ability to understand cause and effect, and the community-based data collection likely produces more error than researcher-controlled efforts. However, given our large sample size and the fact that these errors would not cause a clear bias, we feel these data can substantively contribute to the literature. Our data set includes price information about a limited variety of items and we expect that any given store’s prices fluctuate throughout the year. Our price comparison data set contains information about supermarket chain store prices, which other studies have found to be lower, generally, than other stores. It would be stronger to have comparison price data from a wider variety of store types. The store data we used for comparison, although not an exact match for any of our sample store types, provide a common reference for comparison purposes. Further, we were able to match the comparison store prices to our sampled stores based on county location and month of the year, limiting the potential bias in the relative price analyses related to geographic or temporal differences. However, there is value in presenting findings showing that across all the store types in our study, average produce lowest prices in the study’s low-income neighbourhoods are consistently higher than average supermarket lowest prices matched by county.

While efforts to increase access to fresh fruits and vegetables to all shoppers are important for a variety of reasons, evidence is mounting to suggest that physical access alone is not the answer to the complex problem of dietary and health disparities. Studies repeatedly have demonstrated that consumers, particularly low-income consumers, are price sensitive^(^ ^27^ ^–^ ^29^ ^)^. Our study reveals fruit and vegetable price disparities between low- and higher-income neighbourhoods. Additionally, we find differences in availability, variety, quality and price of produce across store types within low-income neighbourhoods. Our finding that convenience stores on average offer higher-priced, poorer-quality produce than other stores, coupled with the finding that variety is most highly associated with produce price, highlights both the need and the challenge of working in these stores. The vast majority of groceries are not purchased in convenience stores even by low-income shoppers^(^ ^30^ ^,^ ^31^ ^)^. How convenience stores can contribute to improving access to affordable, high-quality produce should be further investigated. Finally, the current study finds that SNAP and WIC participation are associated with better availability, variety and quality of produce, suggesting that these programmes provide an opportunity to continue to improve healthy food access for low-income consumers. Our study examines several possible factors that may influence the food choices made by low-income consumers and suggests that efforts to eliminate dietary disparities and support all residents of the USA to eat a health-promoting diet require a deeper investigation into the underlying issues leading to suboptimal outcomes. In particular, studies are needed to identify what interventions would be most effective for ensuring that quality, affordable, healthy foods are available to all, especially to those with the most limited financial resources.
